# Supplementary material for: Acute Stress Regulates Sex-Related Molecular Responses in the Human Jejunal Mucosa: Implications for Irritable Bowel Syndrome
Source: Cells. 2023 Jan 27;12(3):423. doi: 10.3390/cells12030423 (PMC9913488; doi:10.3390/cells12030423)
Supplement: Supplementary file 1 [file cells-12-00423-s001.zip › cells-2065336-supplementary.pdf]

Supplementary information

Supplementary material and methods:

RNA isolation and cDNA preparation

Biopsies were thawed in ice and the samples were lysed in TRIzol (Invitrogen) with a bead beater (FastPrep mixer, BD medicals), followed by RNA isolation (miRVana kit, Ambion, Invitrogen) and DNase treatment on-column (Qiagen). RNA quantity and quality were confirmed by capillary electrophoresis (Agilent 2100 Bioanalyzer; Agilent Technologies) prior to gene array analysis. Only RNAs with RNA Integrity Number (RIN) >6 was used. Synthesis of cDNA was performed using 1 μg of total RNA with the High-Capacity Reverse Transcription Reagents Kit (Applied Biosystems) following manufacturer's instructions.

RT-qPCR

Expression levels of a selection of genes were analysed by RT-qPCR on an ABI PRISM® 7500 FAST Sequence Detection System (Applied Biosystems) using validated TaqMan Gene Expression Assays (GEA) and cyclophilin as endogenous control (PPIA) (Applied Biosystems). Transcript relative quantification in each sample, including distilled water as negative control, was processed in triplicate. Gene expression was normalized to endogenous genes, and quantified using the comparative Ct method (relative quantification) and the Sequence Detector Software SDS v2.2 (Applied Biosystems). To compare gene expression differences between groups, Ct values for each experimental group was determined involving a target gene and a housekeeping gene (PPIA) that were subtracted to obtain the fold change $2^{-\Delta\Delta Ct}$ ($\Delta\Delta Ct = \Delta Ct_{Post\text{-}stress} - \Delta Ct_{pre\text{-}post}$ = (Ct target $gene_{Post\text{-}stress}$-Ct $PPIA_{Post\text{-}stress}$)-(Ct target $gene_{Pre\text{-}stress}$-Ct $PPIA_{Pre\text{-}stress}$)). For the basal expression levels relative to reference gene (PPIA) levels were calculated as $2^{-\Delta Ct}$ (ΔCt = (Ct target $gene_{Pre\text{-}stress}$-Ct $PPIA_{Pre\text{-}stress}$)) and represented as $\log_{10}$(deltaCt). The expression of genes implicated in epithelial barrier (CLDN1, CLDN2, OCLN, ZO-1, ZO-3), MC function (TRYP and SERPINA1) and stress regulation (NR3C1) were analyzed by RT-qPCR in the jejunal RNA from before and after stress. The complete list of genes assessed and primers used for RT-qPCR is indicated in table 2.

Protein expression by western blot

Equal amounts of protein were separated by electrophoresis and transferred to a polyvinylidene fluoride (PVDF) membrane. Primary antibodies and dilutions used are listed in supplementary Table s2. Peroxidase-conjugated goat anti-rabbit IgG or goat anti-mouse IgG (Pierce) and the chemiluminescence detection system SuperSignal West Femto (Thermo Scientific, Madrid, Spain) were used to detect bound antibodies. Normalization was performed using β-actin as loading control. The bands were scanned and quantitated by densitometry (ImageJ software) and results are

expressed as fold-change of each individual between pre and post-CPS.

Protein expression by immunohistochemistry

N3CR1 expression was assessed through immunohistochemistry using a mouse anti-glucocorticoid receptor monoclonal Ab (Cell Signaling Technology, 47411S). Briefly, tissue sections were incubated with the primary antibody at 1/300 after permeabilization with Triton 0.4% + Tween-20 0.2% in PBS 0.01M, followed by incubation with HRP labelled polymer that is conjugated to secondary antibodies anti-mouse (DAKO). Staining is revealed by incubation with 3,3′-diaminobenzidine (DAB)+substrate-chromogen which results in a brown-coloured precipitate at the antigen site (DAKO EnVision™+ System, HRP). Pictures of whole tissue sections (complete scan of the samples) were taken at 200X with an optical microscope with a coupled camera (LEICA DMLB). The quantification of the staining intensity was carried out with software Fiji (ImageJ).

Supplementary figures:

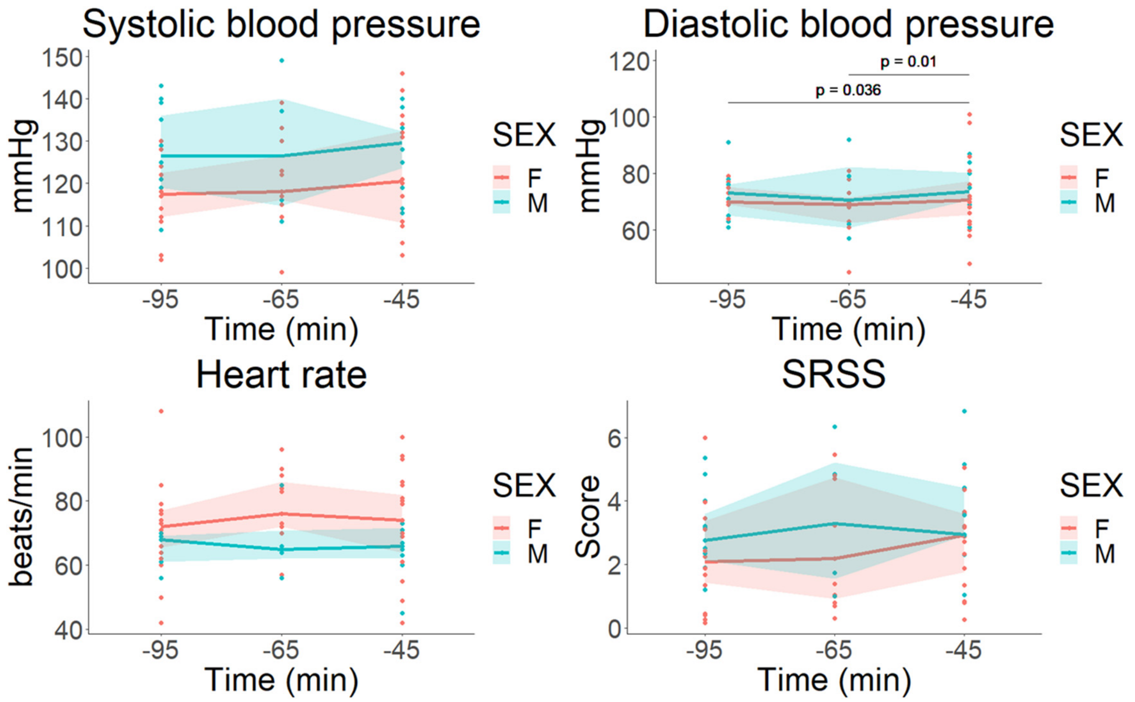

Figure S1. Systemic response to baseline biopsy in subjects submitted to CPS. A Two-way ANOVA revealed differences for Time group in diastolic blood pressure. Lines represent the median for each time point and the area represents the 25 and 75 percentiles min: minutes; SSRS: Subjective Stress Rating Scale.

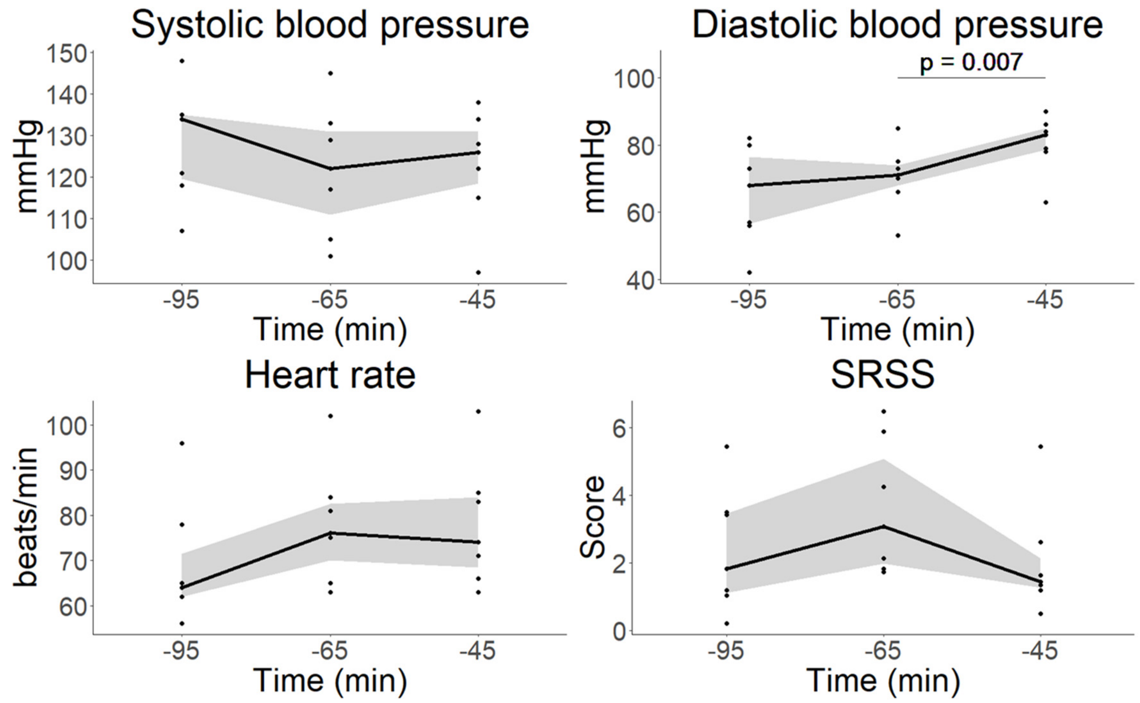

Figure S2. Systemic response to baseline biopsy in subjects submitted to sham stress. A one-way ANOVA revealed differences for Time group in diastolic blood pressure and heart rate. Lines represent the median for each time point and the area represents the 25 and 75 percentiles. min: minutes; SSRS: Subjective Stress Rating Scale.

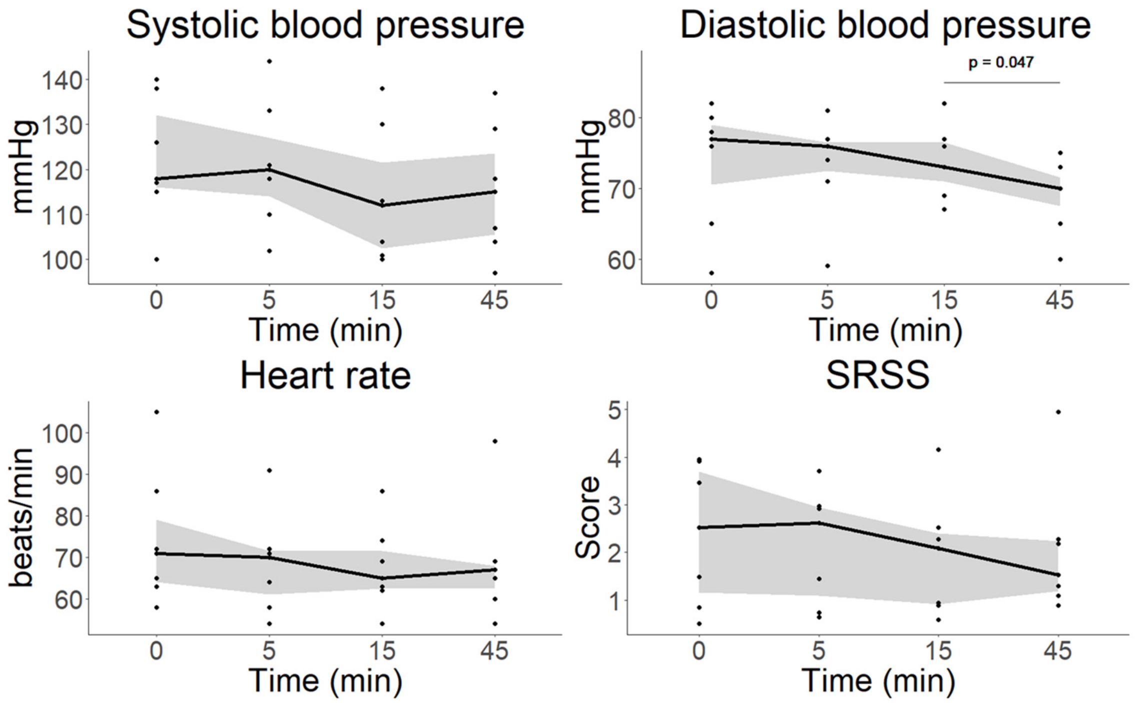

Figure S3. Systemic response to sham stress. A one-way ANOVA revealed significant differences for Time in systolic blood pressure and heart rate. Lines represent the median for each time point and the area represents the 25 and 75 percentiles. min: minutes; SSRS: Subjective Stress Rating Scale.

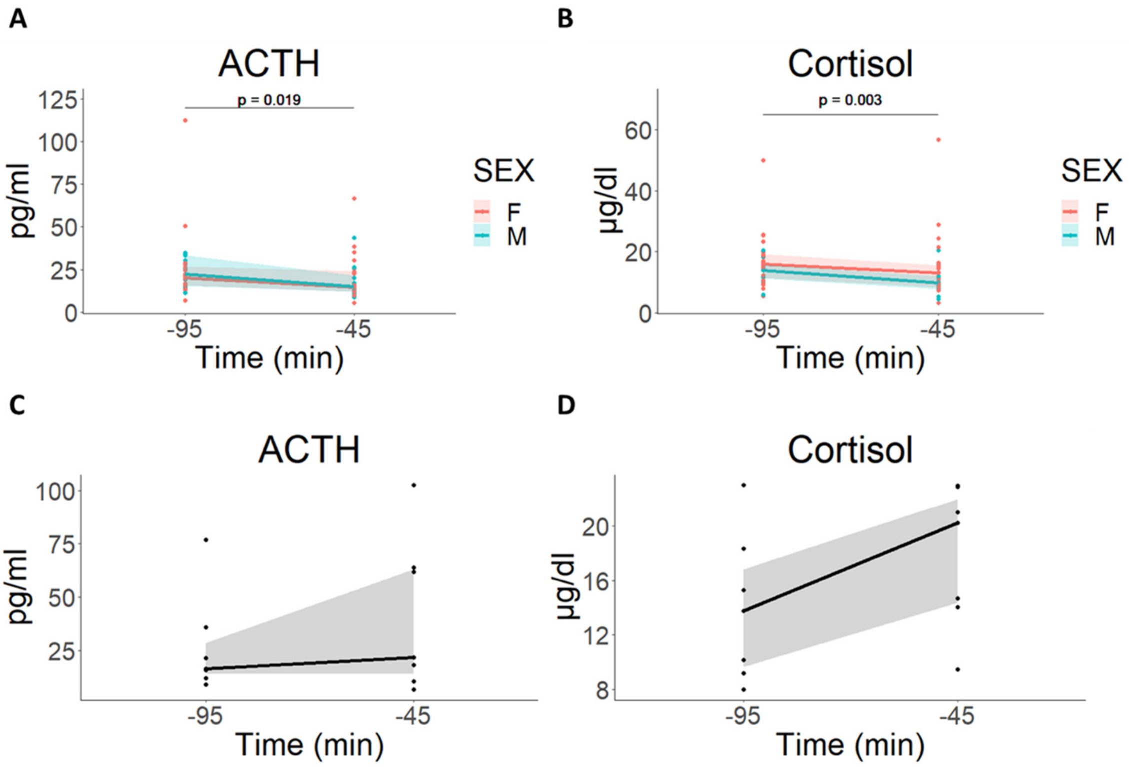

Figure S4. Hormonal response to to baseline biopsy in subjects submitted to CPS (A and B) and sham stress (B and C). (A) ACTH: A Two-way ANOVA revealed significant differences for Time; (B) Cortisol: A Two-way ANOVA revealed significant differences for Time.; (C) ACTH: A one-way ANOVA revealed significant differences for Time (post hoc analysis did not find any significant effect); (D) Cortisol: A one-way ANOVA revealed significant differences for Time (post hoc analysis did not find any significant effect). Lines represent the median for each time point and the area represents the 25 and 75 percentiles. ACTH: adrenocorticotropic hormone; F: females; M: males; min: minutes.

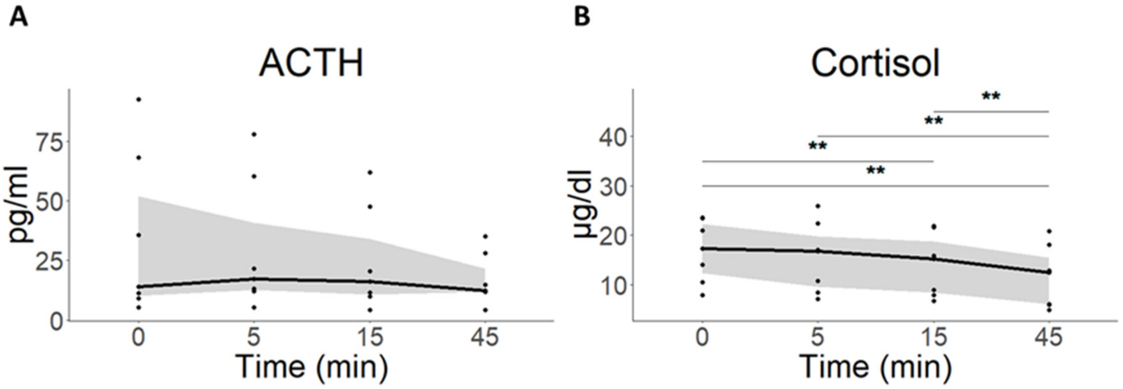

Figure S5. Hormonal response to sham stress. A) ACTH levels and B) Cortisol levels. A one-way ANOVA revealed a significant effect of time group in ACTH and cortisol levels by sham stress (post hoc analysis did nott find any significant effect at ACTH levels). Lines represent the median for each time point and the area represents the 25 and 75 percentile.s ACTH: adrenocorticotropic hormone; F: females; M: males; min: minutes.

A

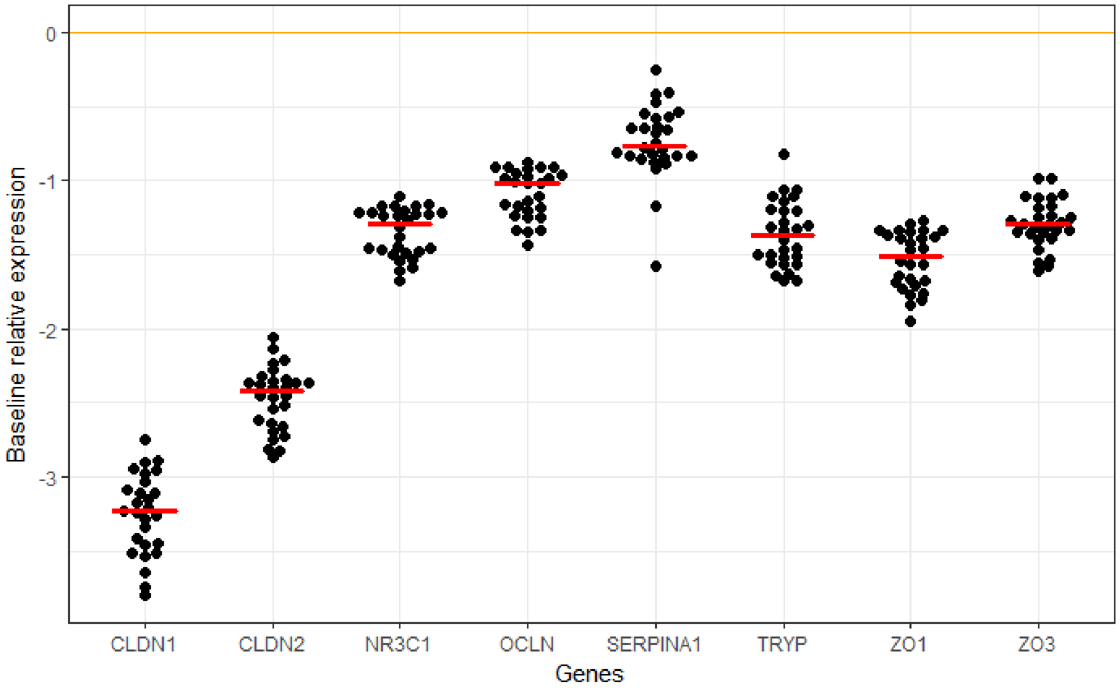

0
-1
-2
-3
Baseline relative expression
CLDN1
CLDN2
NR3C1
OCLN
SERPINA1
TRYP
ZO1
ZO3
Genes

B

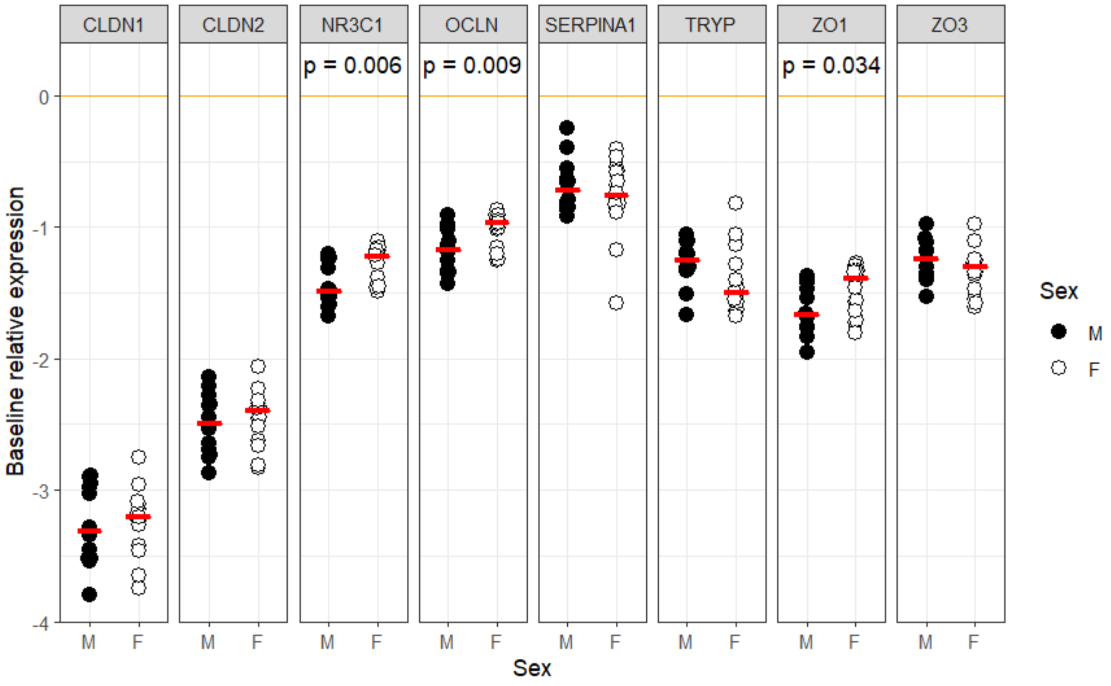

CLDN1
CLDN2
NR3C1
OCLN
SERPINA1
TRYP
ZO1
ZO3
p = 0.006
p = 0.009
p = 0.034
0
-1
-2
-3
-4
Baseline relative expression
M
F
Sex
Sex
M
F

C

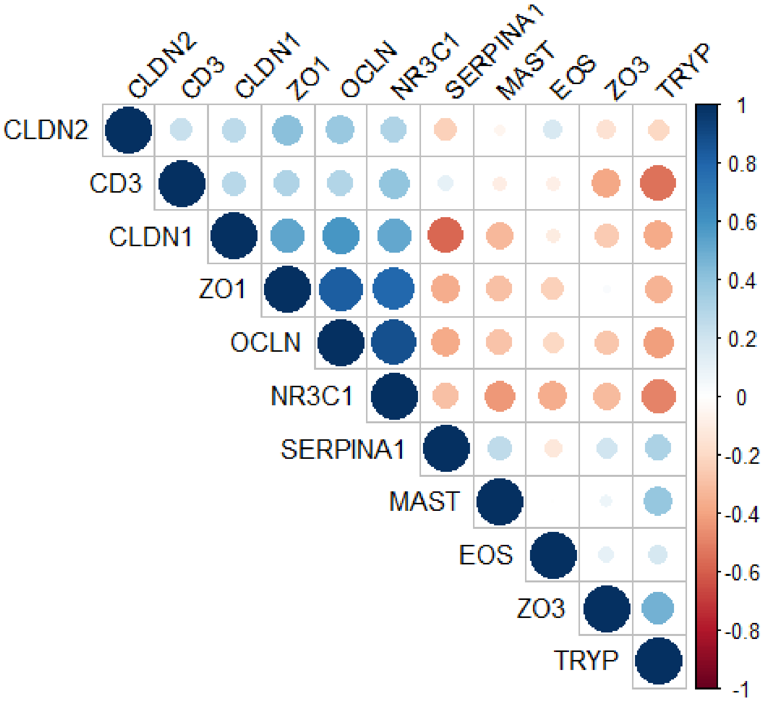

CLDN2
CD3
CLDN1
ZO1
OCLN
NR3C1
SERPINA1
MAST
EOS
ZO3
TRYP
1
0.8
0.6
0.4
0.2
0
-0.2
-0.4
-0.6
-0.8
-1

D

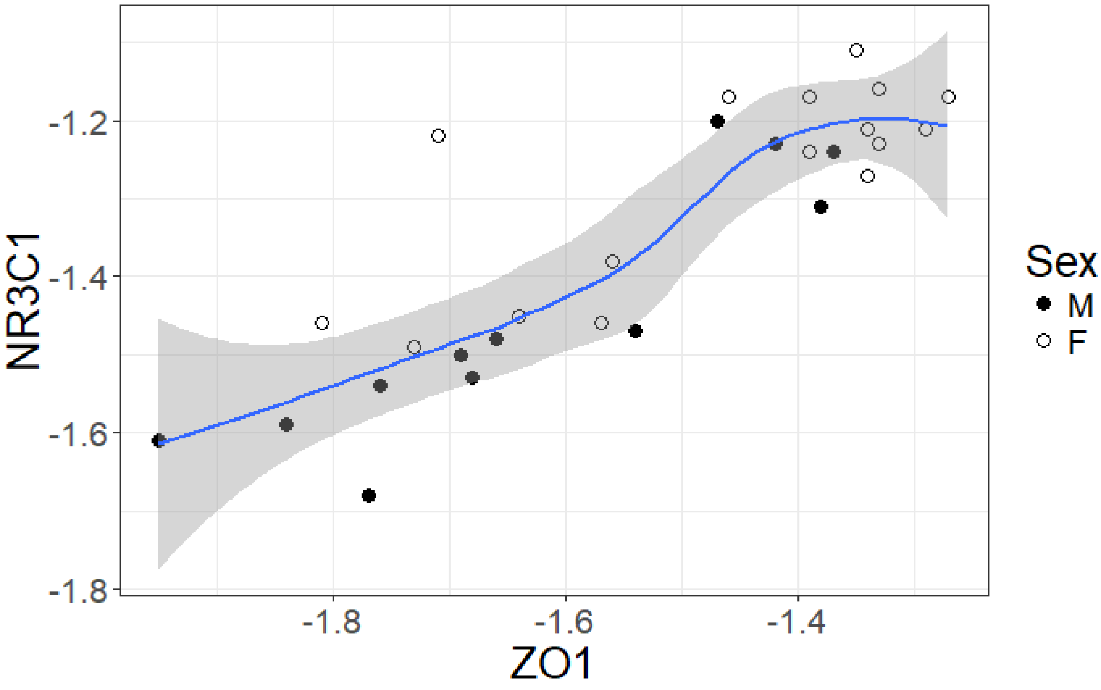

-1.2
-1.4
-1.6
-1.8
NR3C1
-1.8
-1.6
-1.4
ZO1
Sex
M
F

E

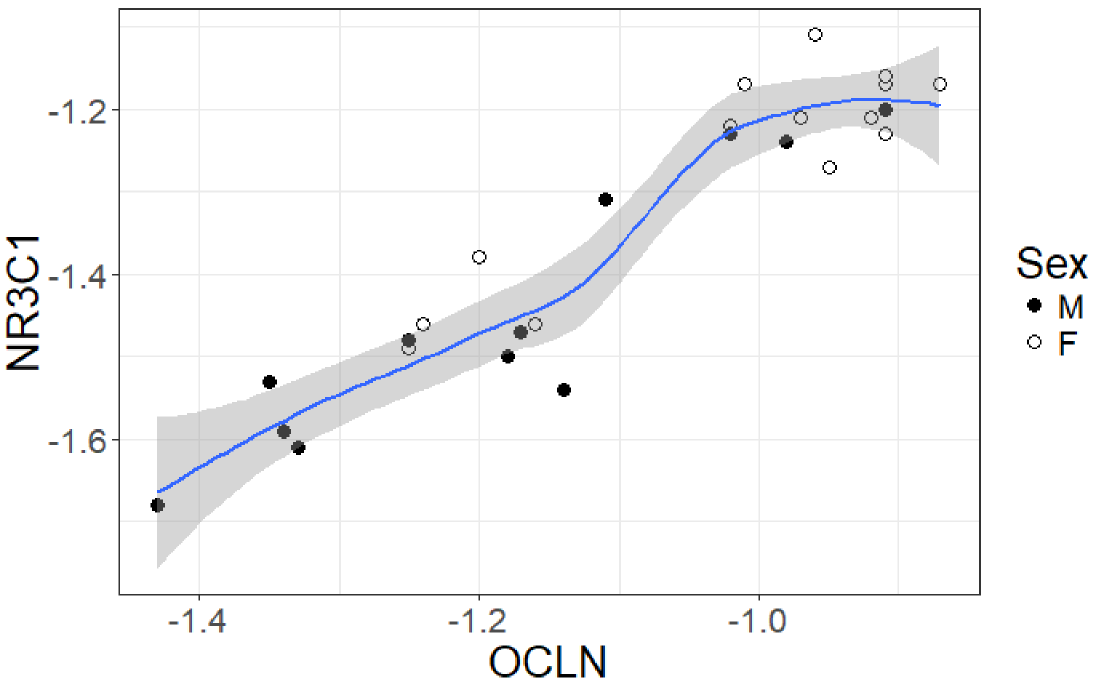

F

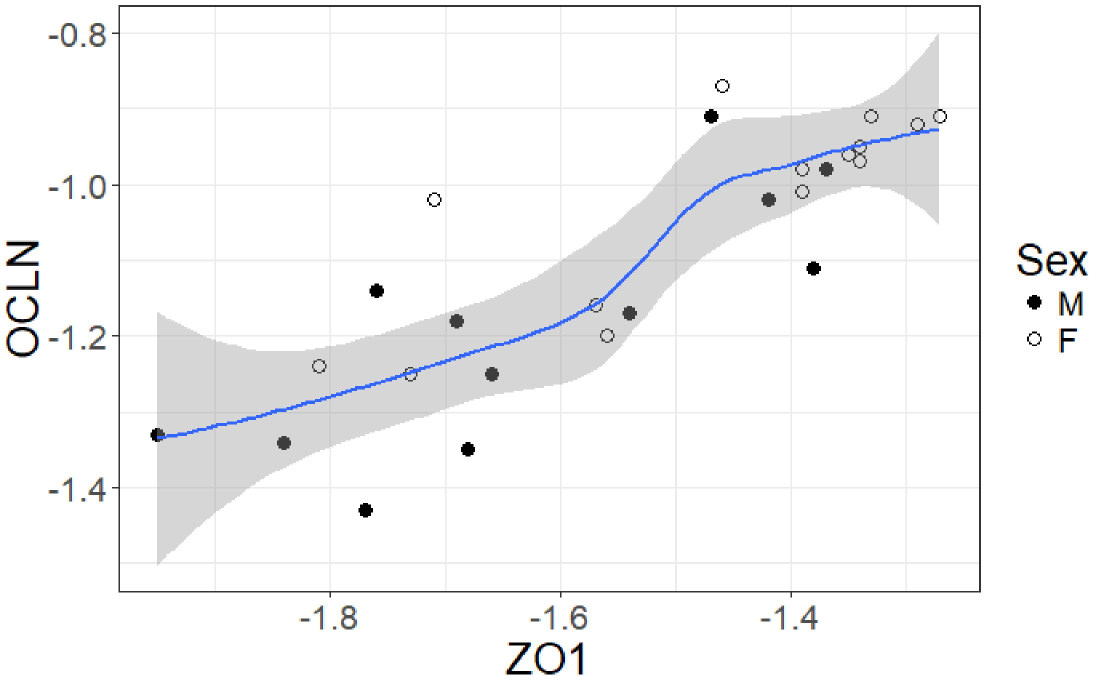

Figure S6: Baseline expression levels of CLDN1, CLDN2, NR3C1, OCLN, Scheme 1. TJP1, TJP3 and TRYP in jejunal mucosa of subjects submitted to CPS. (A) Relative gene expression levels at baseline in the jejunal mucosa relative to the expression level of reference gene. Orange line indicate the expression level of the reference gene (PPIA). The y-axis is represented in $\log_{10}$. The red line indicates the median. (B) Relative gene expression levels at baseline in the jejunal mucosa relative to the expression level of reference gene by sex. Orange line indicate the expression level of the reference gene. The y-axis is represented in $\log_{10}$. The red line indicates the median. (C) Correlation graph with hierarchical clustering of the relative gene expression levels and the

number of MC. The dots size and color of the dots represent the degree of correlation (Spearman's rho). (D-F) Represent the correlation distribution of the relative expression levels among NR3C1, ZO-1 and OCLN. The blue line indicates the Loess regression and the gray area the confidence interval of 95%. Males and females are represented with back circles and white circles, respectively. * Indicates p value < 0.05. CLDN1: claudin 1; CLDN2: claudin 2; OCLN: occludin; ZO-1: zonula occludens 1; ZO-3: zonula occludens 3; TRYP: Tryptase; SERPINA1: Serpin Family A Member 1; NR3C1: glucocorticoid receptor Nuclear Receptor Subfamily 3 Group C Member 1.

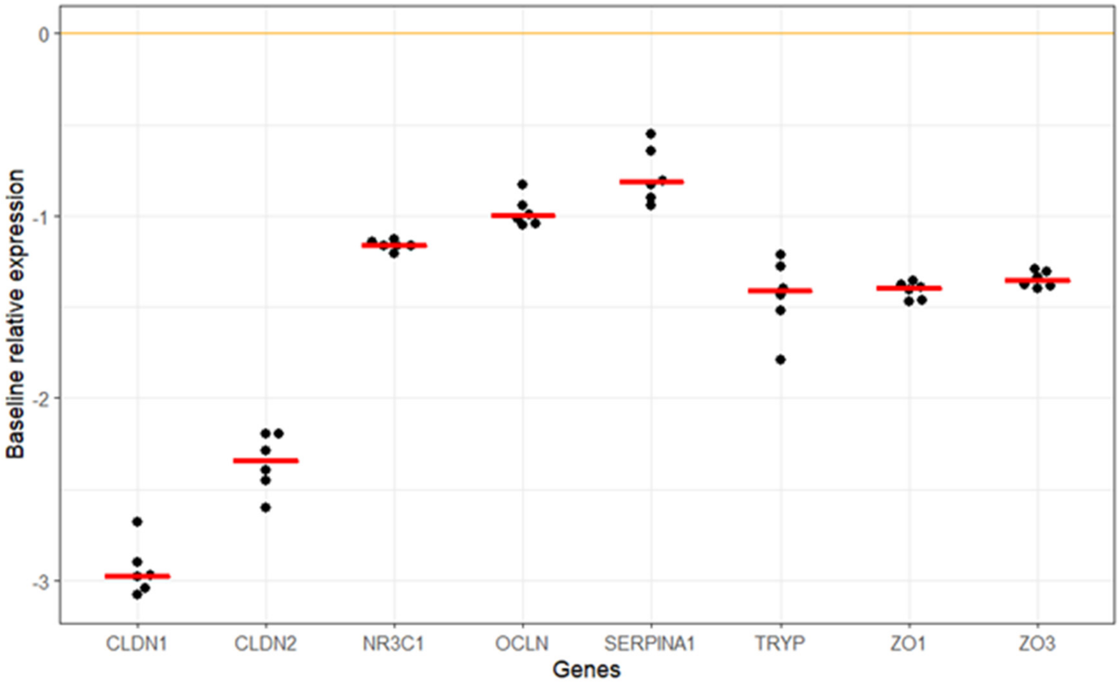

Figure S7. Baseline expression levels of CLDN1, CLDN2, NR3C1, OCLN, SERPINA1, TJP1, TJP3 and TRYP in jejunal mucosa of subjects submitted to sham stress. Relative gene expression levels at baseline in the jejunal mucosa relative to the expression level of reference gene. Orange line indicates the expression level of the reference gene (PPIA). The y-axis is represented in log10. The red line indicates the median. CLDN1: claudin 1; CLDN2: claudin 2; OCLN: occludin; ZO-1: zonula occludens 1; ZO-3: zonula occludens 3; TRYP: Tryptase; SERPINA1: Serpin Family A Member 1; NR3C1: glucocorticoid receptor Nuclear Receptor Subfamily 3 Group C Member 1.

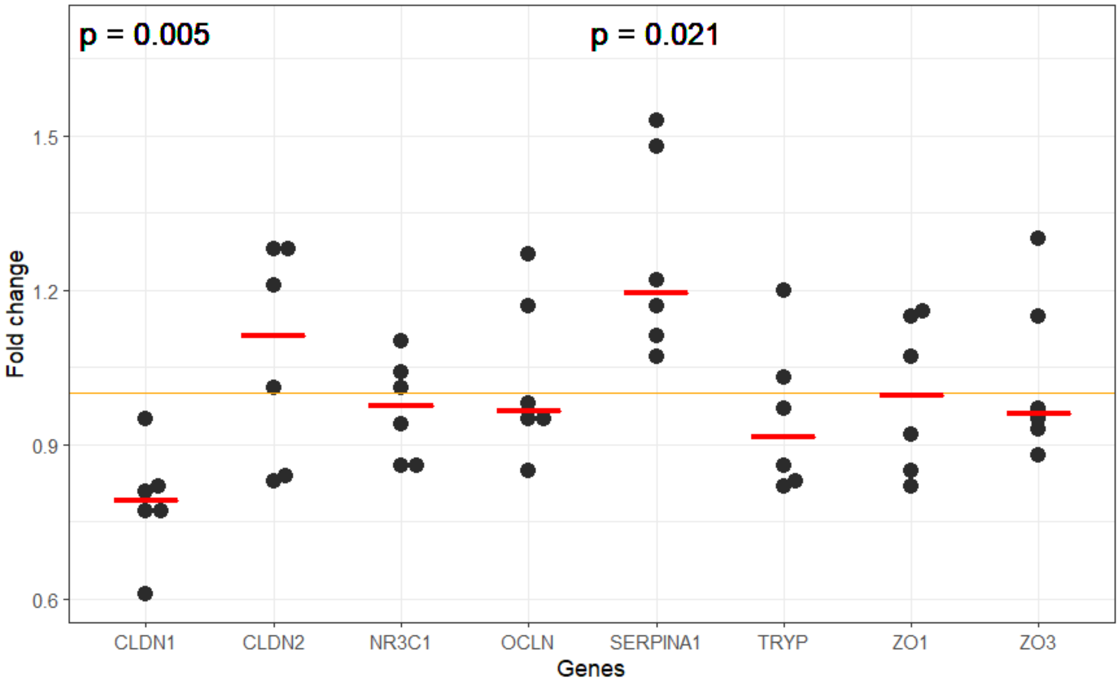

Figure S8: S Gene expression levels ~~changes~~ of CLDN1, CLDN2, NR3C1, OCLN, SERPINA1, ZO-1, ZO-3 and TRYP in jejunal mucosa after sham stress. One-sample t-test, revealed a significant effect on the expression of CLDN1 and SERPINA1. Fold change of gene expression levels induced by stress in jejunal mucosa. The red line indicates the median. CLDN1: claudin 1; CLDN2: claudin 2; OCLN: occludin; ZO-1: zonula occludens 1; ZO-3: zonula occludens 3; TRYP: Tryptase; SERPINA1: Serpin Family A Member 1; NR3C1: glucocorticoid receptor Nuclear Receptor Subfamily 3 Group C Member 1.

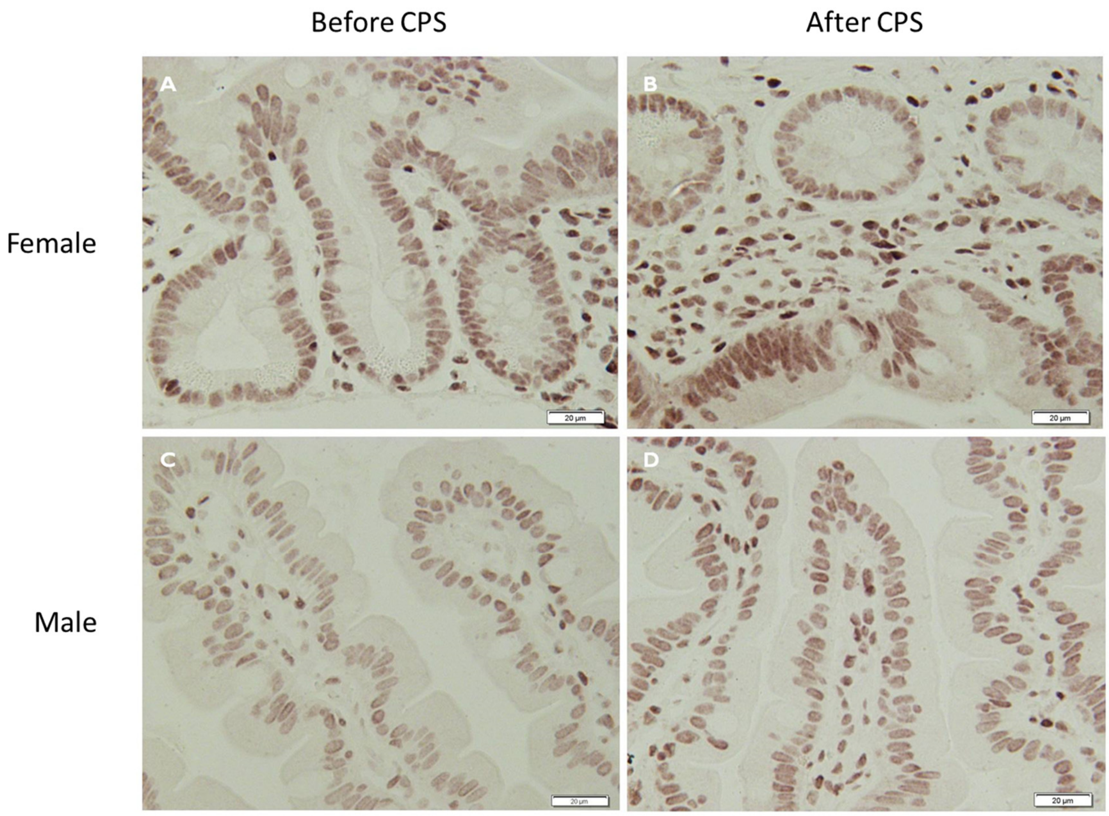

Figure S9: Protein expression levels of NR3C1 in jejunal mucosa after CPS. NR3C1 mean intensity by IHC in jejunal mucosal samples was similar at baseline between male and female groups and we did not find significant modification after CPS in terms of NR3C1 expression and cellular localization. Representative IHCs images showing the NR3C1 staining in the jejunal mucosa of two subjects before (A, C) and after cold pain stress (B, D). CPS: cold pain stress.

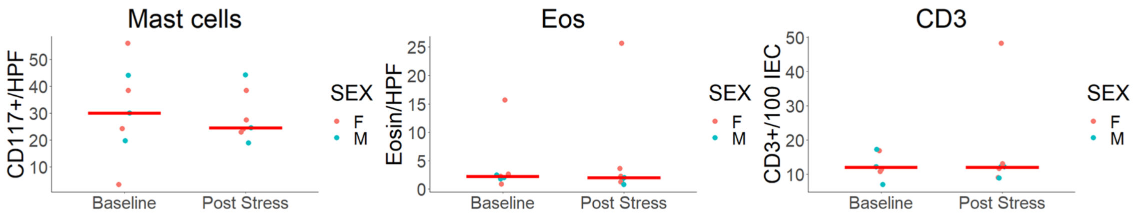

Figure S10. Mucosal inflammation in jejunal mucosa after sham stress. (A) Mast cell counts before (Baseline) and after sham stress (Post Stress) expressed per HPF (400x). (B) Eosinophil counts before (Baseline) and after sham stress (Post Stress) expressed per HPF. (C) Intraepithelial lymphocyte counts before (Baseline) and after sham stress (Post Stress) expressed per 100 intestinal epithelial cells. A one-way Anova did not find significant differences in Time. CD117: cluster of differentiation 117; HPF: high-power field; Eosin: eosinophils; CD3: cluster of differentiation 3; IEC: intestinal epithelial cells; F: females; M: males.

Table S1. List of taqman probes used for the RT-qPCR gene expression assays.

Gene	Gene name	Taqman assay reference number
CLDN1	Claudin 1	Hs 00221623_m1
CLDN2	Claudin 2	Hs 00252666_s1
NR3C1	Nuclear receptor subfamily 3 group C member 1	Hs00353740_m1
OCLN	Occludin	Hs 00170162_m1
PPIA	Peptidylprolyl isomerase A (cyclophilin A)	Hs 99999904_m1
SERPINA1	Serpin family A member 1	Hs00165475_m1
TPSAB1	Tryptase beta 2 (gene/pseudogene) (TRYP in the main text and figures)	Hs 02576518_Gh
ZO1	Tight junction protein 1 or zonula occludens 1	Hs 00268480_m1
ZO3	Tight junction protein 3 or zonula occludens 3	Hs 00274276_m1

Table S2. Primary Antibodies used for Western blot and IHC studies.

Protein	Antibody	Supplier (ref)	Dilution/Incubation	Technique
**Occludin**	Polyclonal rabbit anti-human Occludin	Invitrogen(71-1500)	1/1000 (ON, 4ºC)	WB
**ZO-1**	Monoclonal mouse anti-human ZO-1	Invitrogen (33-9100)	1/250 (ON, 4ºC)	WB
**β-actin**	Monoclonal mouse anti-human β-actin	Sigma-Aldrich (A5441)	1/10000 (30 min, RT)	WB
**CD3**	Monoclonal rabbit anti-human CD3	Ventana (790-4341)	ready to use (32 min, RT)	IHC
**CD117**	Monoclonal rabbit anti-human CD117	Ventana(790-2951)	ready to use (32 min, RT)	IHC
**NR3C1**	Monoclonal Mouse anti-glucocorticoid receptor	Cell Signaling Technology (47411S)	1/300 (ON, 4ºC)	IHC

WB: Western blot; IHC: Immunohistochemisty.

Table S3. Clinical variables from subjects submitted to Sham stress.

	Sex		
	**Male**	**Female**	**p value**
**Variable**	M [Q1-Q3]	F [Q1-Q3]	
**Age (years)**	26.1 [20.4.2-30.2]	24.3 [21.8-32.4]	0.64
**Holmes-Rahe's scale (score)**	45 [41-158]	100 [60-130.5]	0.13
**Cohen's scale (score)**	14 [11-20]	9.5 [6.5-19]	0.32
**Beck's inventory (score)**	0 [0-1.8]	0 [0-3]	0.67

Table legend: Holmes-Rahe's Scale (to assess the level of stress over the last year) Reference values: low stress <150), moderate 150-299, high ≥300. Cohen's Scale (to assess the level of stress over the last month). reference values: no perceived stress <19), mild perceived stress 19-28), moderate perceived stress 29-38, severe perceived stress >38). Beck's Depression Inventory, to evaluate the level of depression during the last week. Reference values: no depression <10, mild 10-18, moderate 19-29, severe depression 30-63. Data are expressed as median with first and third quartiles [Q1-Q3] A Mann-Whitney U test was used for comparisons of continuous variables between groups.
